# Engineering of recombinant *Escherichia coli* cells co-expressing poly-γ-glutamic acid (γ-PGA) synthetase and glutamate racemase for differential yielding of γ-PGA

**DOI:** 10.1111/1751-7915.12075

**Published:** 2013-08-06

**Authors:** Mingfeng Cao, Weitao Geng, Wei Zhang, Jibin Sun, Shufang Wang, Jun Feng, Ping Zheng, Anna Jiang, Cunjiang Song

**Affiliations:** 1 Key Laboratory of Molecular Microbiology and Technology for Ministry of Education, Nankai UniversityTianjin, 300071, China; 2 Tianjin Institute of Industrial Biotechnology, Chinese Academy of SciencesTianjin, 300308, China; 3 State Key Laboratory of Medicinal Chemical Biology, Nankai UniversityTianjin, 300071, China

## Abstract

Poly-γ-glutamic acid (γ-PGA) is a promising environmental-friendly material with outstanding water solubility, biocompatibility and degradability. However, it is tough to determine the relationship between functional synthetic enzyme and the strains' yield or substrate dependency. We cloned γ-PGA synthetase genes *pgsBCA* and glutamate racemase gene *racE* from both L-glutamate-dependent γ-PGA-producing *Bacillus licheniformis* NK-03 and L-glutamate-independent *B. amyloliquefaciens* LL3 strains. The deduced RacE and PgsA from the two strains shared the identity of 84.5% and 78.53%, while PgsB and PgsC possessed greater similarity with 93.13% and 93.96%. The induced co-expression of *pgsBCA* and *racE* showed that the engineered *Escherichia coli* strains had the capacity of synthesizing γ-PGA, and LL3 derived PgsBCA had higher catalytic activity and enhanced productivity than NK-03 in Luria–Bertani medium containing glucose or L-glutamate. However, the differential effect was weakened when providing sufficient immediateness L-glutamate substrate, that is, the supply of substrate could be served as the ascendance upon γ-PGA production. Furthermore, RacE integration could enhance γ-PGA yield through improving the preferred d-glutamate content. This is the first report about co-expression of *pgsBCA* and *racE* from the two *Bacillus* strains, which will be of great value for the determination of the biosynthetic mechanism of γ-PGA.

## Introduction

Poly-γ-glutamic acid (γ-PGA) is a naturally occurring polyanionic polypeptide that consists of repeating units of D- and L-glutamic acid via amide linkages between α-amino and γ-carboxyl groups (Ashiuchi and Misono, 2002). The molecular weight of microbial γ-PGA varies from 10k to 10 000k and the stereochemical structure includes three types: a homopolymer of d-glutamic acid, a homopolymer of L-glutamic acid, and copolymer of random combinations of D-/L-glutamic acid (γ-DL-PGA) (Ashiuchi and Misono, 2003). With the chiral centre existing in glutamate unit and the abundant active sites of carboxylic groups present in the main chain, γ-PGA and its derivatives were endowed with outstanding water solubility, biocompatibility and degradability, and have been successfully utilized in hydrogels, humectants, flocculants, thickeners, dispersants, cryoprotectants, drug carriers, and cosmetic and biological food additives (Shih and Van, 2001; Sung *et al*., 2005).

The microorganisms, capable of producing γ-PGA and drawing the molecules as capsule protective components and extracellular nutritious secretion, mainly belong to Gram-positive bacteria, such as *Bacillus* genus, *Staphylococcus epidermidis* (Kocianova *et al*., 2005), Archaeobacteria species *Natronococcus occultus* (Niemetz *et al*., 1997) and *Natrialba aegyptiaca* (Hezayen *et al*., 2000). However, Candela and colleagues (2009) classified the first Gram-negative bacterium, *Fusobacterium nucleatum*, which demonstrated to produce γ-PGA as interaction factor for dental plaque formation. γ-PGA-producing strains are divided into two types: one produces γ-PGA in the presence of L-glutamate in medium, and the other does not (Ito *et al*., 1996). Most of the known γ-PGA producers, such as *B*. *subtilis* (*natto*) IFO 3335 (Goto and Kunioka, 1992), *B*. *subtilis* chungkookjang (Ashiuchi *et al*., 2001), *B. licheniformis* ATCC9945a (Gardner and Troy, 1979) and *B*. *licheniformis* NK-03 (Cao *et al*., 2010), belong to the former group, whereas for the latter group, just a few strains have been characterized, including *B. subtilis* TAM-4 (Ito *et al*., 1996), *B. licheniformis* A35 (Cheng *et al*., 1989) and *B. amyloliquefaciens* LL3 (Cao *et al*., 2011). Although γ-PGA producers with high productivity and industrial applications are mostly L-glutamic acid-dependent strains, it is of great interest to choose L-glutamic acid-independent producers for the studies on γ-PGA biosynthesis mechanism and its development potential because of the lower cost and simplified downstream processing.

To date, heterologous expression of γ-PGA synthetase genes (*pgsBCA*) from L-glutamic acid-dependent strains have been achieved in various organisms (Ashiuchi *et al*., 1999; 2001; Jiang *et al*., 2006). It was previously reported that tobacco leaf cells (Tarui *et al*., 2005) and the glutamate-producing *Corynebacterium glutamicum* strains harbouring *pgsBCA* expression vectors could display γ-PGA synthetase and accumulate extracellular γ-PGA without L-glutamate (Sung *et al*., 2003; Cao *et al*., 2010). Cao *et al*. (2011) reported the successful expression of *pgsBCA* genes from *B. amyloliquefaciens* LL3 in *Escherichia coli* JM109, resulting in the production of γ-PGA without L-glutamate. However, it would be more significant if the differential expression of *pgsBCA* genes from these two strains was demonstrated. Furthermore, glutamate racemase (*racE*) can be overexpressed to control the stereochemical composition of D-/L-glutamate leading to an increase in the production of γ-PGA, as well as the proportion of d-glutamate present in the polymer. It was revealed that overexpression of *glr* (*racE*) gene not only increased the production of γ-PGA but also increased the proportion of d-glutamate in γ-PGA (Ashiuchi *et al*., 1999; Jiang *et al*., 2011).

In this study, the *pgsBCA* genes and glutamate racemase gene (*racE*) from L-glutamic acid-dependent strain *B*. *licheniformis* NK-03 and L-glutamic acid-independent strain *B. amyloliquefaciens* LL3 were cloned and expressed in *E*. *coli* JM109. The differential expression was carried out in the medium containing either L-glutamate or glucose as carbon source. The extraction products were characterized by the weighing yields and fraction ratios of D-/L-isomer of γ-PGA monomer using reversed-phase high-performance liquid chromatography (reversed-phase HPLC). The results herein can supply the clue to the comparable functional structure of synthetase and be used to elucidate the molecular catalytic mechanism and the stereochemical modulation in γ-PGA biosynthesis.

## Results and discussion

### Cloning and alignment of *pgsBCA* genes from *Bacillus* strains

As expected for representative γ-PGA-producing strains, *B*. *licheniformis* NK-03 and *B*. *amyloliquefaciens* LL3 were capable of synthetizing γ-PGA with different molecular weight in the presence or absence L-glutamate, respectively. It is known that the γ-PGA synthetase complex consists of three functional subunits (PgsB, PgsC and PgsA) and is responsible for catalysing glutamate to synthesize γ-PGA with the type of membranous adenosine triphosphate (ATP)-dependent amide-ligase (Ashiuchi *et al*., 2004; Wang *et al*., 2011). Therefore, the cloning and sequence alignment of *pgsBCA* genes will be fundamental to understand the molecular mechanism of γ-PGA synthesis.

**Figure 1:**
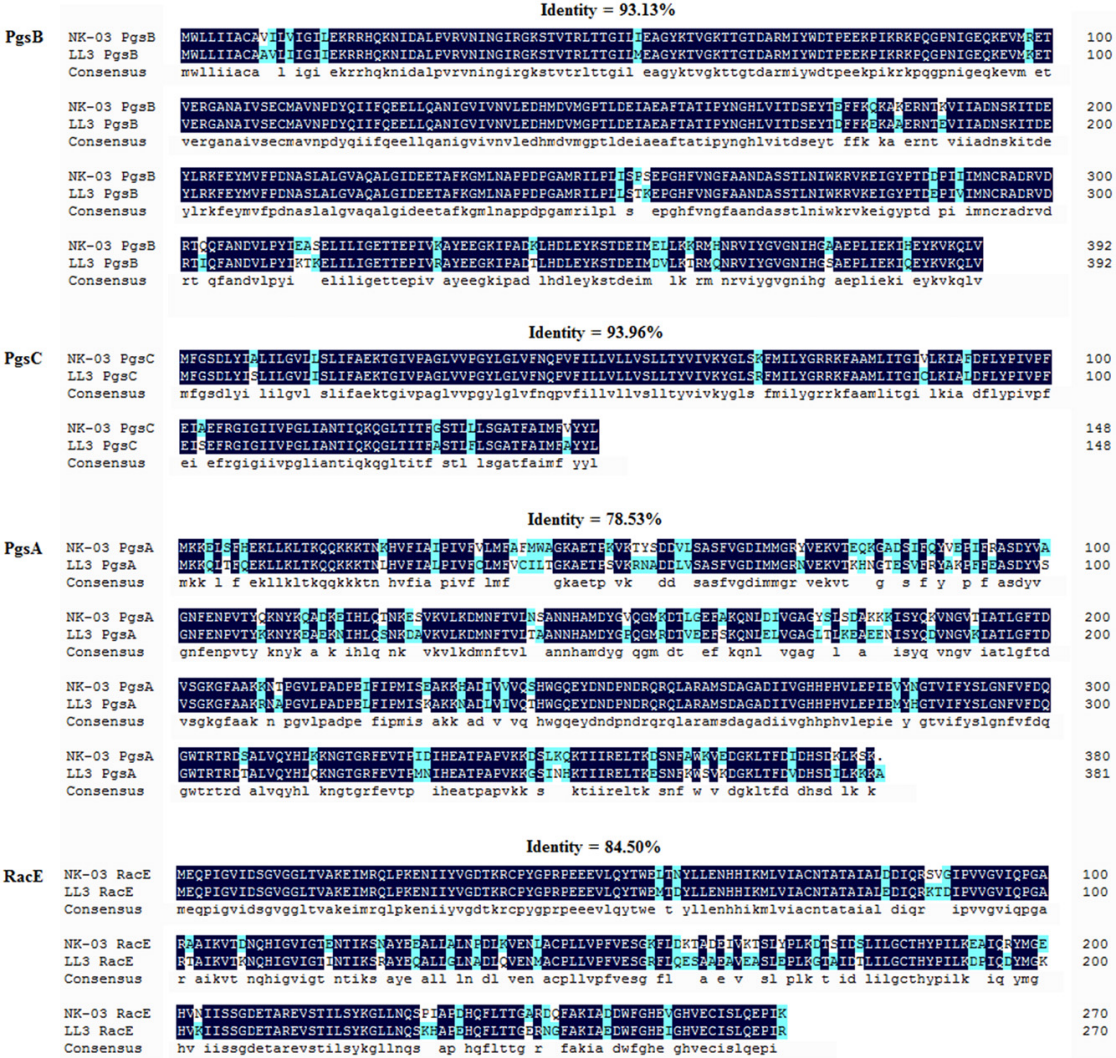
Sequence alignment of amino acid sequences of γ-PGA synthetase complex (PgsBCA) and glutamate racemase (RacE) from *B. licheniformis* NK-03 and *B. amyloliquefaciens* LL3. The residues with identity are represented by lower case beneath the sequences.

The sequence of *pgsBCA* was amplified from genome DNA of NK-03 and LL3 strains as before (Cao *et al*., 2010; 2011), which was about 3000 bp and contained three open-reading frames (ORFs), designated as *pgsB*, *pgsC* and *pgsA*. After alignment by BLAST (Basic Local Alignment Search Tool) and DNAMAN, the synthetase constituents PgsB and PgsC showed great similarity above 93%, while the PgsA showed only about 78.5% homology (Table S1). Based on the consensus of PgsBCA components and their amino acids sequences (Fig. 1), the feature of each element have been analysed and validated as that reported (Ashiuchi and Misono, 2003). It was revealed that PgsB conserves the consensus sequences found in enzymes of an amide ligase superfamily (Eveland *et al*., 1997). It harbours an ATP-binding motif at the N-teminal residues of 37–42 (GIRGKS), which is responsible for catalysing the hydrolysis of essential ATP, providing the energy for γ-PGA synthesis. PgsC, the most conserved component of PgsBCA complex, is a hydrophobic and a membrane-bound protein containing four transmembrane helices regions. The active site of PgsBCA, which was supposed to be constituted by PgsB and PgsC, was assumed to display ATPase activity and assimilate both isomers of γ-DL-PGA as the substrate (Urushibata *et al*., 2002). As the core structure for membrane integration and the transporter of γ-PGA, PgsA consisted mainly of hydrophobic and cationic amino acid residues, and showed the most variable part of synthetase to generate the differential elongation (molecular weight) of γ-PGA (Candela and Fouet, 2006). However, to elucidate the precise function of each membrane-associated component in γ-PGA polymerization and transportation, it should be recurred to three-dimensional modelling and crystal structure resolution.

### Characterization of *racE* gene from *Bacillus* strains

RacE, the glutamate racemase, was universally acknowledged as the primary enzyme of d-glutamate conversion from its enantiomer L-glutamate in the dynamic kinetic resolution of γ-PGA synthesis (Ashiuchi *et al*., 2001). The *racE* gene was cloned into pMD 19-Simple T vector and sequenced; in addition, the deduced amino acid sequence was aligned using DNAMAN software. It was revealed that the ORFs of *racE* gene from NK-03 and LL3 has size of 816 bp, and were deposited into GenBank accession nos. GQ375411 and GQ375412, respectively. The *racE* gene encodes 271 amino acids, the initial codon of which was TTG but not ATG. According to the deduced amino acid analysis, the RacE was estimated to have a molecular mass of 30 kDa, and the 12 amino acid sequence (MEQPIGVIDSGV) in its N-terminal region was determined to be Edman degradation region (Fig. 1). Furthermore, the regions surrounding the two cysteine residues (Cys-73 and Cys-184) are highly conserved; glutamate racemase reactions are proposed to proceed through a two-base mechanism involving the two essential cysteine residues (Gallo *et al*., 1993; Doublet *et al*., 1996; Ashiuchi *et al*., 1998). As it was reported that racemase from *Bacillus* was inactivated by 2-nitro-5-thiocyanatobenzoate, the two conserved cysteine residues are deemed to play an important role in the catalysis (Ashiuchi *et al*., 1998).

As shown in Fig. 1, the homology score of the glutamate racemase between *B*. *licheniformis* NK-03 and *B*. *amyloliquefaciens* LL3 was about 84.5%, and it was completely identical at the N-terminal region, composed by 55 amino acids. Because of the γ-PGA products from the two strains had the similar low content of d-glutamate (less than 2%), we hypothesized that the N-terminal sequence may act as the binding domain of L-glutamate accumulated in cells and the enhancer of the racemization. In addition, the initial codon of TTG presents low translation efficiency, which brought about the low activity of RacE and then the low content of d-glutamate in γ-PGA products. Ashiuchi and colleagues (1998) cloned the *glr* (*racE*) gene from *B*. *subtilis* IFO 3336 and overproduced it in the soluble fraction of the *E*. *coli* clone cells with the initial codon substitution of ATG for TTG. The cloned enzyme showed similar properties to those of the racemase from *B. subtilis* IFO 3336, suggesting that the enzyme spontaneously and effectively folds to become active in the *E. coli* overproducer. Therefore, the initial TTG of *racE* gene from NK-03 and LL3 was replaced with ATG to produce a large amount of the gene products from the recombinant cells of *E*. *coli* in the following co-expression of *pgsBCA* and *racE*.

### Construction of co-expression pTrcNRP and pTrcLRP vectors

The 3.0 kb size of *pgsBCA* genes from *B*. *licheniformis* NK-03 and *B*. *amyloliquefaciens* LL3 were fused between BamHI-HindIII sites of pTrc99A (size of 4.2 kb), constructing recombinant plasmid that were named pTrcNpgs and pTrcLgs (size of 7.2 kb), respectively. Subsequently, the 0.8 kb *racE* gene holding KpnI-BamHI restriction sites was inserted into the foregoing site of *pgsBCA* located after the *trc* promoter, incorporating co-expression vectors pTrcNRP and pTrcLRP (size of 8.0 kb). *Escherichia coli* JM109 clones that harboured pTrc99A-*pgsBCA*-*racE* were selected by the methods of colony polymerase chain reaction (PCR) and recombinant plasmid enzyme digestion.

**Figure 2:**
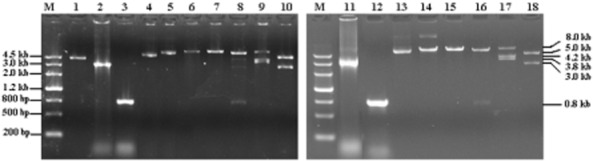
Electrophoresis analysis profile of recombinant plasmid pTrcLRP and pTrcNRP in *E. coli* JM109. Lane 1–10, test of pTrc99A harbouring LL3 *pgsBCA* and *racE* genes; Lane 11–18, test of pTrc99A harbouring NK-03 *pgsBCA* and *racE* genes. *Note:* Lane M: Tiangen Marker III (Tiangen); Lane 1: pTrc99A/BamHI (4.2 kb); Lane 2: LL3 *pgsBCA* (3.0 kb); Lane 3: LL3 *racE* (0.8 kb); Lane 4: pTrcLRP (Supercoil, 8.0 kb); Lane 5: pTrcLpgs/BamHI (7.2 kb); Lane 6: pTrcLRP/KpnI (8.0 kb); Lane 7: pTrcLRP/BamHI (8.0 kb); Lane 8: pTrcLRP/KpnI+BamHI (7.2 and 0.8 kb); Lane 9: pTrcLRP/KpnI+HindIII (4.2 and 3.8 kb); Lane 10: pTrcLRP/BamHI+HindIII (5.0 and 3.0 kb); Lane 11: NK-03 *pgsBCA* (3.0 kb); Lane 12: NK-03 *racE* (0.8 kb); Lane 13: pTrcNpgs/BamHI (7.2 kb); Lane 14: pTrcNRP/KpnI (8.0 kb); Lane 15: pTrcNRP/BamHI (8.0 kb); Lane 16: pTrcNRP/KpnI+BamHI (7.2 and 0.8 kb); Lane 17: pTrcNRP/KpnI+HindIII (4.2 and 3.8 kb); Lane 18: pTrcNRP/BamHI+HindIII (5.0 and 3.0 kb).

Judging from the bands migration distance compared with Marker III (Tiangen) in 0.8% agarose gel electrophoresis shown in Fig. 2, it was revealed that the fusion expression vectors pTrcLRP and pTrcNRP were successfully introduced into *E*. *coli* JM109. To our knowledge, the PgsBCA synthetase and glutamate racemase (RacE) genes in recombinant strains that could synthesize γ-PGA were only from *B. subtilis* strains (Ashiuchi *et al*., 1999). Therefore, the related work about *pgsBCA* and *racE* cloned from *B. licheniformis* and *B. amyloliquefaciens* strains and transformed into *E. coli* will enrich the co-expression systems and supply the clue for differential synthesis of γ-PGA as well.

### Determination of co-expression of *racE* and *pgsBCA* genes in *E. coli*

**Figure 3:**
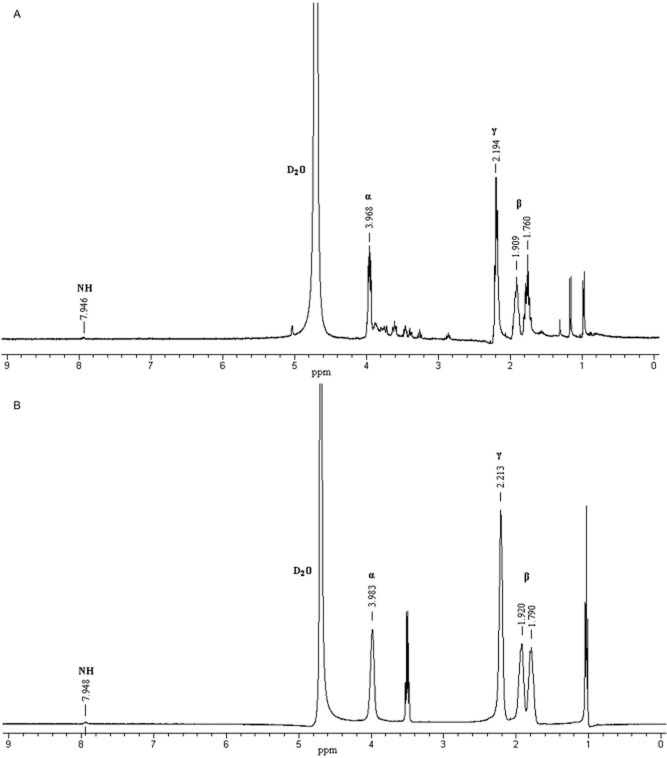
^1^H NMR spectrum of γ-PGA produced from engineered 109LRP and 109NRP. A. ^1^H NMR spectrum of γ-PGA from 109LRP product in D_2_O. The chemical shifts of α-CH (3.968 ppm), β-CH_2_ (1.76 and 1.909 ppm), γ-CH_2_ (2.194 ppm) and N-H (7.946) were labelled in the peaks location. B. ^1^H NMR spectrum of γ-PGA from 109NRP product in D_2_O. The chemical shifts of α-CH (3.983 ppm), β-CH_2_ (1.790 and 1.920 ppm), γ-CH_2_ (2.213 ppm) and N-H (7.948) were labelled in the peaks location.

The recombinant *E. coli* JM109 strains, harbouring pTrcLpgs, pTrcNpgs, pTrcLRP and pTrcNRP were designated as 109LP, 109NP, 109LRP and109NRP, respectively (Table 1). The flask culture of Luria–Bertani (LB) plus 2% carbon source, metal ions (Mg^2+^ and Mn^2+^) suggested that the recombinant strains had the capacity of synthesizing γ-PGA. The ^1^H NMR spectrum of purified products revealed that the chemical shifts of α-CH, β-CH_2_, γ-CH_2_ and N-H are nearly overlapped with that of Sigma γ-PGA standard and the origin strains of LL3 and NK-03 (Cao *et al*., 2010; 2011). As shown in Fig. 3, the peak positions of γ-PGA from 109LRP and 109NRP were displayed as follows: α-CH (3.968/3.983 ppm), β-CH_2_ (1.760/1.790 ppm and 1.909/1.920 ppm), γ-CH_2_ (2.194/2.213 ppm) and N-H (7.946/7.948 ppm). In addition, the products were further determined for amino acid analysis by reversed phase HPLC and thin layer chromatography (data not shown). The results suggest that the fusion of *racE* and *pgsBCA* genes were successfully expressed, and γ-PGA was the primary product of the four engineered strains. However, it was surprising that there was only an obscure band corresponding to a molecular mass of 30 kDa (RacE), yet no clear band of target protein with molecular mass of 43 kDa (PgsB), 16 kDa (PgsC) and 42 kDa (PgsA) observed by sodium dodecyl sulfate-polyacrylamide gel electrophoresis (SDS-PAGE) (data not shown). As we know, the PgsB, PgsC and PgsA parts constitute the active form of the synthetase complex, which is generally anchored into *Bacillus* cell membrane. It is conjectured that the different hydrophobicity and permeability of cell membrane structure between *E. coli* and *Bacillus* would bring about the inaccurate location and incapable function of PgsBCA, then following the scarce quantity for electrophoresis analysis. Considering there was no reports about the enzymatic purification and characterization of PgsBCA complex, this study will be employed to interpret the instability of membranous complex and supply a promising approach to obtain the crystallized amount of PgsBCA.

**Table 1:** Strains and plasmids used in this study

Stain or plasmid	Relevant characteristics	Reference or source
Plasmids		
pMD 19-Simple T vector	T/A-cloning vector; *lacZ*; *Amp*^r^	TaKaRa (Dalian)
pTrc99A	Cloning vector, *ori* from pBR322, *trc* promoter, *Amp*^r^	Amann and colleagues (1988)
pTrcLpgs	pTrc99A carrying *pgsBCA* genes from LL3 strain	This study
pTrcNpgs	pTrc99A carrying *pgsBCA* genes from NK-03 strain	This study
pTrcLRP	pTrc99A carrying *racE* and *pgsBCA* genes from LL3 strain	This study
pTrcNRP	pTrc99A carrying *racE* and *pgsBCA* genes from NK-03 strain	This study
Strains		
*B. licheniformis* NK-03	Wild type, L-glutamic acid dependent producer of γ-PGA	Laboratory stock
*B. amyloliquefaciens* LL3	Wild type, L-glutamic acid independent producer of γ-PGA	Laboratory stock
*E. coli* strains		
JM109	*recA supE*44 *endA*1 *hsdR*17 *gyrA*96*relA*1 *thi* △(*lac-proAB*) *F*′ (*traD*36 *proAB*+ *lacI^q^lacZ*△M15)	TaKaRa (Dalian)
109LP	JM109 harbouring vector pTrcLpgs	This study
109NP	JM109 harbouring vector pTrcNpgs	This study
109LRP	JM109 harbouring vector pTrcLRP	This study
109NRP	JM109 harbouring vector pTrcNRP	This study

### Fermentation and characterization of γ-PGA produced by engineered strains

A previous study showed that the synthetase encoded by *pgsBCA* from *B. amyloliquefaciens* LL3 could produce γ-PGA in engineered *E. coli* with the de novo pathway from glucose, and the yield was resembled to that of the recombinant *C. glutamicum* strain harbouring *pgsBCA* from *B. licheniformis* NK-03 (Cao *et al*., 2011). However, the PgsBCA activity and productivity differences between NK-03 and LL3, following with the possible molecular catalysis mechanism of L-glutamic acid-dependent and -independent γ-PGA-producing strains, remained undetermined. Furthermore, addition of Mn^2+^, cooperated with Mg^2+^, to the polymer-synthesis medium of *E. coli* cells co-expression genetic system harbouring *pgsBCA* and *racE* genes could increase the yield and d-glutamate content of γ-PGA, which could be adopted in the fermentation process of γ-PGA (Ashiuchi *et al*., 1999; Jiang *et al*., 2011).

The resultant cultivation showed that the four engineered strains grew almost synchronously and could produce γ-PGA in both glucose and L-glutamate medium after 24 h of induction. It was interesting that regardless of the harbouring vector, the yield of γ-PGA produced by LL3 *pgsBCA* was higher than that of NK-03; in other words, the LL3-derived PgsBCA appeared to have greater catalytic activity. As shown in Table 2, 109LP and 109LRP could synthesize 22% and 48% higher yields of γ-PGA than 109NP (0.308 ± 0.025 g l^−1^) and 109NRP (0.349 ± 0.016 g l^−1^), respectively, in glucose medium, while it was not so distinctive when the strains cultivated in L-glutamate medium. However, the molecular weight of γ-PGA produced by 109LP and 109LRP in both glucose and L-glutamate medium possessed lower *M*_w_ than 109NP and 109NRP, which had almost the same tendency as that of the original LL3 (*M*_w_ = 470 kDa) and NK03 (*M*_w_ = 1360 kDa). These observations suggested that the catalytic efficiency of PgsBCA from LL3 could really outperform that from NK-03, while the elongation dynamics was affected by the separate subunit of the synthetase complex. In addition, the activity bias could be offset by supplying enough substrate of L-glutamate, i.e. the divergence of PgsBCA did not contribute very well to the glutamic acid-independent γ-PGA-producing strains, while the glutamate synthase likely played an important part in γ-PGA synthesis.

**Table 2:** Characterization of γ-PGA produced by the four *E. coli* recombinant strains

Strains	Yield (g l^−1^)	D-glutamate content (%)	Molecular weight (× 10^4^Da)
*E. coli*-pTrcLpgs (109LP)^a^	0.376 ± 0.021	2.46 ± 0.32	3.23 ± 0.26
*E. coli*-pTrcNpgs (109NP)^a^	0.308 ± 0.025	3.28 ± 0.27	3.74 ± 0.38
*E. coli*-pTrcLRP (109LRP)^a^	0.517 ± 0.027	8.96 ± 0.53	5.09 ± 0.33
*E. coli*-pTrcNRP (109NRP)^a^	0.349 ± 0.016	9.33 ± 0.47	5.83 ± 0.42
*E. coli*-pTrcLpgs (109LP)^b^	0.558 ± 0.018	3.01 ± 0.45	5.35 ± 0.28
*E. coli*-pTrcNpgs (109NP)^b^	0.533 ± 0.022	5.94 ± 0.66	5.89 ± 0.55
*E. coli*-pTrcLRP (109LRP)^b^	0.645 ± 0.016	18.13 ± 1.36	6.23 ± 0.46
*E. coli*-pTrcNRP (109NRP)^b^	0.603 ± 0.033	19.59 ± 0.98	6.95 ± 0.79

a Carbon source of glucose.

b Substrate of L-glutamate.

As the glutamate racemase could supply the preferred d-glutamate substrate for γ-PGA polymerization, we estimated that the d-monomer content and productivity of γ-PGA should be improved significantly in 109LRP and 109NRP versus that of the parent strains. The results from the γ-PGA yield and d-glutamate content measurement using reversed phase HPLC (Fig. S1) showed great consistency with our hypothesis in either glucose or L-glutamate medium. When using L-glutamate as substrate, the d-monomer content in recombinant 109LRP (18.13%) and 109NRP (19.45%) strains was much higher than that of the 109LP (3.01%), 109NP (5.94%), and the original LL3 (1.5%) and NK-03 (2.0%) *Bacillus* strains, which was due to the regulation of RacE caused by the initial codon substitution of ATG for TTG. Meanwhile, the racemization capacity of RacE from LL3 was conjectured to be better than that of NK-03, owing to the elevated racemization level of RacE of sixfold and threefold, respectively. The similar result of d-monomer content of γ-PGA was acquired in glucose medium (Table 2), while the improvement of γ-PGA yield from 109LRP was much higher than that from 109NRP, which may be attributed to the stronger coordination ability of L-glutamate synthesis and RacE catalysis reaction. Furthermore, the molecular weight was increased as the yield and d-monomer content of γ-PGA, which was evidently affected by the synergy between RacE and PgsBCA. At present, the detailed works on stereochemical composition control of *Bacillus* γ-PGA regulated by RacE are effectively carried out through the combinatorial test and overexpression, including the directed-sites mutation of *racE* gene, which would supply the two species with the needs of different applications.

Although the co-expression of *racE* and *pgsBCA* had successfully presented an appreciable productivity in glucose medium (without L-glutamate), compared with that of other reported engineered *E. coli* strains harbouring only *pgsBCA* genes (Ashiuchi *et al*., 1999; Jiang *et al*., 2011; Wang *et al*., 2011), the optimized production of γ-PGA by the candidate 109LRP in flask culture was just 1.26 g l^−1^, which was still not as high as the wild type of *Bacillus* strains (Cao *et al*., 2010; 2011). Therefore, lots of works will be done to regulate the composition and the biosynthesis of γ-PGA, including the synergism and combinational performances of the concentration of substrate, the stability and permeability of cell membrane, and the activities of glutamate synthase, glutamate dehydrogenase, glutamate racemase, γ-PGA synthetase and depolymerase. Worth to be mentioned, the genome sequence of *B. amyloliquefaciens* LL3 has been completed and deposited into GenBank with accession no. CP002634 (Geng *et al*., 2011); the genome of *B. licheniformis* NK-03 is under sequencing. The future focus will be on systematical analysis of functional genes and regulatory elements screened from the grand data of proteomics and metabolomics. Furthermore, the ongoing crystal structure resolution, and rational designs of glutamate and γ-PGA metabolic pathways' enzymes will provide us in-depth understanding of the biochemical and molecular mechanism of γ-PGA synthesis.

## Experimental procedures

### Bacterial strains, plasmids and culture conditions

The bacterial strains and plasmids used in this study are listed in Table 1. *Bacillus* and *E. coli* JM109 as the hosts for cloning and expression were routinely cultivated aerobically on LB (10 g tryptone, 5 g yeast extract, 10 g NaCl per litre of water) solid or liquid medium at 37°C. The media were supplemented with the following carbon sources, metal ions and antibiotics when required: 2% L-glutamate or glucose, 0.5% MgCl_2_, 0.05% MnSO_4_ and 60 μg ml^−1^ of filter sterilized ampicillin (Amp). Samples were regularly taken for measurement of optical density at 600 nm (OD_600_) and γ-PGA.

### Reagents and kits

The DNA polymerases (rTaq, Ex-Taq and LA-Taq), restriction enzymes and DNA ligase solution were supplied by TaKaRa Biotech (Dalian, China). All oligonucletide primers used in this study (Table 3) were ordered from BGI Genomics (Shenzhen, China), and DNA sequencing was performed by Tianyi Huiyuan (Beijing, China). Extraction and purification of DNA samples were accomplished by Tiangen DNA gel purification kit, plasmid extraction kit and DNA Marker III (Beijing, China). All chemicals and standards were purchased from Sigma (Shanghai, China).

**Table 3:** Primers used in this study

Primer	Sequence (5′-3′)^a^	Function
Npgs-F	CGCGGATCCAAGGAGATGTCGAAAGCAATGT	NK-03 *pgsBCA* cloning
Npgs-R	CCCCAAGCTTCATCTTTATCACTCCGTTTATT	
Lpgs-F	CGCGGATCCAGAAGGAGATGTCAAAAATCAATG	LL3 *pgsBCA* cloning
Lpgs-R	CCCAAGCTTGATTTTCATTTGTTTTTCACTCCGC	
NracE-F	CGGGGTACCGAGGCGATTTTGATGGAAC	NK-03 *racE* cloning
NracE-R	CGCGGATCCACTATCTTTTAATCGGTTCTT	
LracE-F	CGGGGTACCGAGGCGATTTTGATGGAAC	LL3 *racE* cloning
LracE-R	CGCGGATCCAACAGCGGGTTTTTTGATTTAT	

a The restriction sites are underlined.

### Cloning and alignment of genes encoding γ-PGA synthetase and glutamate racemase

Routine DNA manipulations were carried out as described by Sambrook and Russell (2001). The *pgsBCA* genes responsible for γ-PGA synthesis were amplified from chromosomal DNA of *B. licheniformis* NK-03 and *B. amyloliquefaciens* LL3 using the restriction sites (forward: BamHI, reverse: HindIII) incorporated primers Npgs-F/Npgs-R and Lpgs-F/Lpgs-R. TA-cloning was processed with the purified PCR product and pMD 19-Simple T vector (TaKaRa), the positive clones of which were screened by colony PCR and enzyme digestion. Subsequently, the inserted fragments from positive clones were sequenced, and the ORFs were identified using the National Center for Biotechnology Information ORF finder tool and DNASTAR software (DNASTAR, Inc., Madison, WI, USA). The alignments of nucleotides and amino acid sequences between NK-03 and LL3 were accomplished by using BlastN and DNAMAN software (Lynnon Co., Pointe-Claire, QC, Canada). Likewise, the previous manipulations of *pgsBCA* could be employed to perform the cloning and intercomparsion of the glutamate racemase gene *racE* and the related coding enzyme. However, the difference was that the restriction sites were KpnI in forward primer (NracE-F and LracE-F) and BamHI in reverse primer (NracE-R and LracE-R), respectively.

### Plasmid construction for expression of *racE* and *pgsBCA* genes

Based on the deduced ORFs, the sequenced fragments of *pgsBCA* from L-glutamic acid-dependent producer NK-03 or L-glutamic acid-independent producer LL3 were digested with BamHI-HindIII from T-vector and then subcloned into the same sites of pTrc99A to generate the PgsBCA expression vector pTrcNpgs or pTrcLpgs, respectively. In succession, the *racE* gene was excised from T-vector by KpnI-BamHI and fused in the foregoing site of *pgsBCA*, i.e. in the direction of *trc* promoter of pTrc99A, incorporating PgsBCA and RacE co-expression vectors pTrcNRP and pTrcLRP. The positive clones were tested by colony PCR and recombinant plasmid enzyme digestion.

### Co-expression of *racE* and *pgsBCA* in *E. coli*

Strains 109LP, 109NP, 109LRP and109NRP were obtained by transforming *E. coli* JM109 competent cells with pTrcNpgs, pTrcLpgs, pTrcNRP and pTrcLRP, respectively (Table 1). The recombinant cells were selected on LB plates supplemented with Amp (100 μg ml^−1^) and were subsequently inoculated into 5 ml LB broth containing Amp (60 μg ml^−1^). After overnight shaking cultivation at 37°C (200 rpm), 1% (v/v) inoculum was transferred into 500 ml conical flasks containing 100 ml LB medium plus 2% L-glutamate or glucose, 0.5% MgCl_2_, 0.05% MnSO_4_ and 60 μg ml^−1^ of Amp. The optimal expression conditions of *trc* promoter for fusion genes of *racE* and *pgsBCA* were obtained when mid-log-phase cells (OD_600_ = 1.0) were induced with 1 mM isopropyl-β-D-thiogalactopyranoside (IPTG) at 28°C for 24 h with stirring.

The resulting broth was separated by centrifugation for 15 min at 10 000 *g*. The supernatant was used for γ-PGA separation and purification, while the harvested cells were gathered for protein analysis by SDS-PAGE with linear gradient of gel concentration from 8∼12%. The cells were resuspended in 0.1 M sodium phosphate buffer (pH 7.4) and disrupted by sonication in ice-cold buffer of 50 mM Tris-HCl (pH 8.0), 150 mM NaCl, 10 mM dithiothreitol. The supernatant liquid and sedimentation pellets were collected selectively and boiled for denaturation with the electrophoresis detection of potential RacE and PgsBCA.

### Purification and characterization of γ-PGA

γ-PGA was recovered and purified by a modified precipitation method reported previously (Cao *et al*., 2011). The supernatant of IPTG-induced broth was poured into fourfold volumes of cold anhydrous ethanol; then, the precipitate was centrifuged at 20 000 *g* (20 min, 4°C) and dissolved in deionized water. The γ-PGA-containing solution was dialysed against ice-cold Milli-Q water in a dialysis membrane bag (molecular weight cut-off = 8000–14 000) for 24 h at 4°C, and the remain was lyophilized (−50°C) to obtain the purified biopolymer.

The ^1^H NMR spectroscopy was carried out to identify products of γ-PGA, using the heavy water (D_2_O)-dissolved samples scanned in nuclear magnetic resonance spectrometer (Varian Infinity plus 400, Varian Inc., Palo Alto, CA, USA), and the fingerprints were compared with a standard γ-PGA (Sigma). To determine the ratios of L- and d-isomer of γ-PGA hydrolysate, reversed phase HPLC was employed according to the FDAA (Marfey's reagent) precolumn derivation method with an Alltech GRACE C18 column (Alltech Associates, Inc., Deerfield, IL, USA) (Cao *et al*., 2010). The molecular weight of the products was measured by gel permeation chromatography using the eluent of 0.25 M NaNO_3_ in Alltech system controller equipped with a Shodex KW804 column (Showa Denko KK, Tokyo, Japan) and Schambeck SFD refractive index detector (Schambeck SFD GmbH, Bad Honnef, Germany) (Cao *et al*., 2011).

## Supporting information

**Fig. S1.** d-glutamate content measurement of γ-PGA produced by the four *E. coli* recombinant strains using the reversed phase HPLC method.

**Table S1.** The comparison of PgsBCA synthetase complex and RacE from NK-03 and LL3.
