# Supplementary material for: Engineering of recombinant Escherichia coli cells co-expressing poly-γ-glutamic acid (γ-PGA) synthetase and glutamate racemase for differential yielding of γ-PGA
Source: Microb Biotechnol. 2013 Aug 6;6(6):675–84. doi: 10.1111/1751-7915.12075 (PMC3815934; doi:10.1111/1751-7915.12075)
Supplement: Supplementary file 1 [file mbt0006-0675-SD1.doc]

**Supporting information**

**Table S1** The comparison of PgsBCA synthetase complex and RacE from NK-03 and LL3

| Sequence alignment | *B*. *amyloliquefaciens* LL3 (%) | | | | | | | |
| --- | --- | --- | --- | --- | --- | --- | --- | --- |
|  | *pgsB* | *pgsC* | *pgsA* | *racE* | PgsB | PgsC | PgsA | RacE |
| *B*. *licheniformis* NK-03 (%) | 81.73 | 83.11 | 73.80 | 76.59 | 93.13 | 93.96 | 78.53 | 84.50 |

**
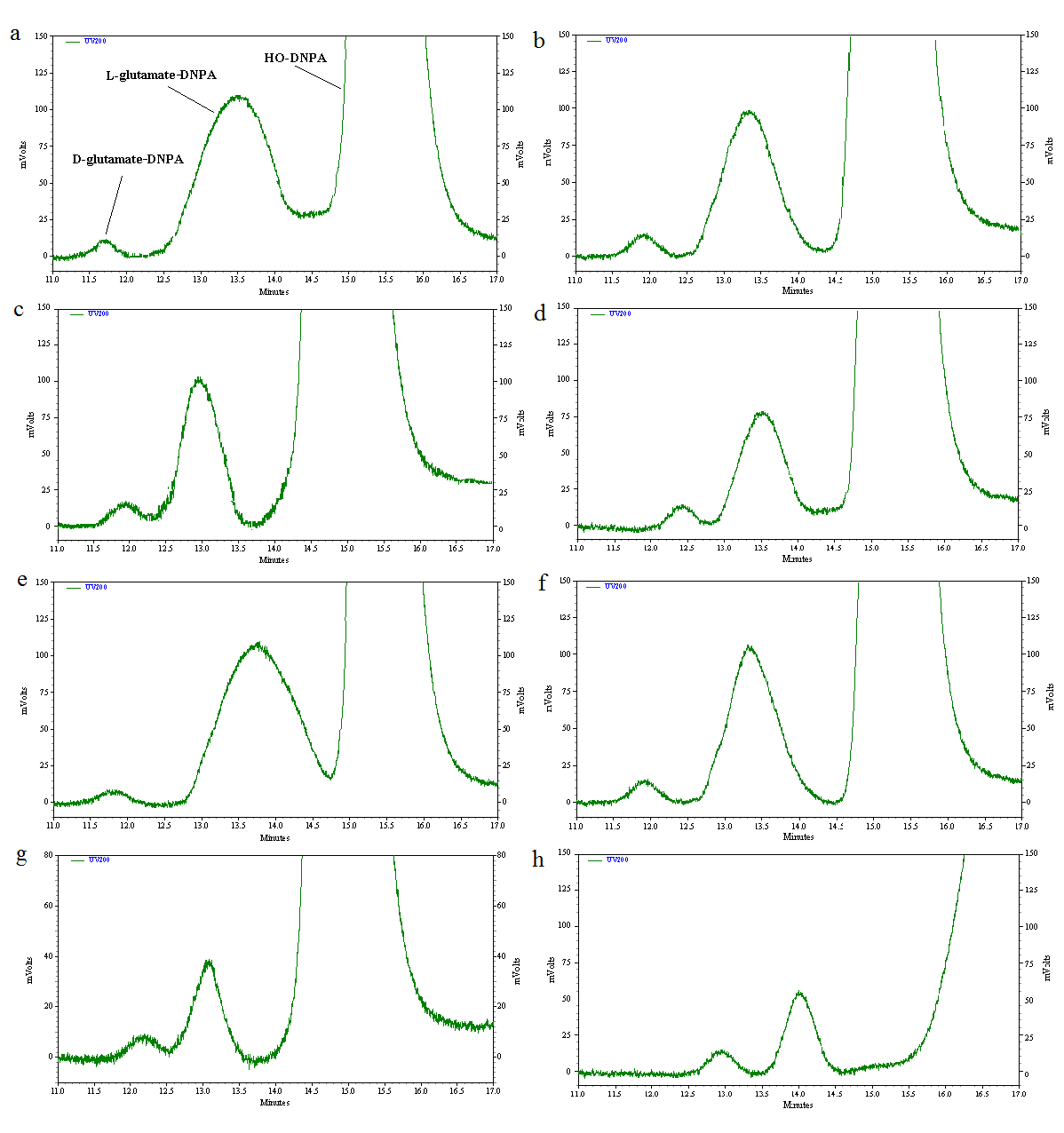
**

**Fig. S1** D-glutamate content measurement of γ-PGA produced by the four *E. coli* recombinant strains

using the reversed phase HPLC method

Note: **(a)** The spectrum of D-glutamate content of γ-PGA produced by 109LP in medium containing glucose; **(b)** The spectrum of D-glutamate content of γ-PGA produced by 109NP in medium containing glucose; **(c)** The spectrum of D-glutamate content of γ-PGA produced by 109LRP in medium containing glucose; **(d)** The spectrum of D-glutamate content of γ-PGA produced by 109NRP in medium containing glucose; **(e)** The spectrum of D-glutamate content of γ-PGA produced by 109LP in medium containing L-glutamate; **(f)** The spectrum of D-glutamate content of γ-PGA produced by 109NP in medium containing L-glutamate; **(g)** The spectrum of D-glutamate content of γ-PGA produced by 109LRP in medium containing L-glutamate; **(h)** The spectrum of D-glutamate content of γ-PGA produced by 109NRP in medium containing L-glutamate
